# Learning with technology in physiotherapy education: design, implementation and evaluation of a flipped classroom teaching approach

**DOI:** 10.1186/s12909-019-1728-2

**Published:** 2019-07-31

**Authors:** Yngve Røe, Michael Rowe, Nina B. Ødegaard, Hilde Sylliaas, Tone Dahl-Michelsen

**Affiliations:** 1 Department of Physiotherapy, Faculty of Health Sciences, OsloMet – Oslo Metropolitan University, Oslo, Norway; 2 Faculty of Community and Health Sciences, University of Western Cape, Cape Town, South Africa

**Keywords:** Flipped classroom, Teaching with technology, Physiotherapy education

## Abstract

**Background:**

The purpose of the study was to describe the design, implementation and evaluation of a flipped classroom teaching approach in physiotherapy education. The flipped classroom is a blended learning approach in which students receive digital lectures as homework, while active learning activities are used in the classroom. Flipped classroom teaching enables a learning environment that aims to develop higher-order cognitive skills.

**Methods:**

The study design was a historically controlled, prospective, cohort study. An eight week theoretical course on musculoskeletal disorders was redesigned, moving from a conventional approach to a flipped classroom model. Pre-class learning material consisted of about 12 h of video lectures and other digital learning resources that were split up over the duration of the course. In-class activities consisted of seven full-day seminars where students worked in groups in order to solve problem-based assignments. The assignments were designed to reflect authentic clinical problems and required critical thinking and reasoning. Outcomes were measured with course-grades and compared with historical controls of conventional teaching, using descriptive statistics. Self-perceived learning outcomes and students’ experiences were also collected in a survey.

**Results:**

Fifty-one students passed the course exam, two failed and one did not attend (*n* = 54). The share of students with Excellent, Very good and Good (ABC) performances increased by more than 10% relative to any previous year. In addition, Satisfactory, Sufficient and Failed performances (DEF) decreased by more than 10%. Almost two thirds of the students preferred the flipped classroom approach as compared with conventional teaching. Interaction with peers and educators, and flexibility, were the most positive factors that were reported by students. Long seminars, time-constraints and low motivation with respect to preparation and educators’ roles were the most common complaints.

**Conclusions:**

A flipped classroom approach in physiotherapy education resulted in improved student performances in this professional programme, when compared with conventional teaching. Students responded positively to the collaborative learning environment, especially with respect to the associated autonomy and flexibility. There were indicators that all groups did not work optimally and that accountability to other group members did not always ensure pre-classroom preparations.

## Background

The flipped classroom is a blended learning approach in which students receive digital lectures as homework, while active learning activities are used in the classroom [1–3]. The rationale is that the students’ preparation prior to class enhances the efficacy of the learning activities. Findings of a systematic review on the use of flipped classroom in higher education, indicate improved student satisfaction and increased academic performance, as measured by improved examination results, pre-test to post-test scores and course grades, compared with conventional teaching [4]. Furthermore, there is also evidence that flipped classroom teaching increases class-attendance [4].

With respect to health education, a recent meta-analyses on the effectiveness of the flipped classroom concluded that the approach yielded a statistically significant improvement in learner performance when compared with conventional teaching [5]. A similar systematic review in higher education nursing programmes yielded academic outcomes that were either neutral or positive [6]. The authors of the review concluded that more studies on implementation, evaluation and refinement of the flipped classroom in health education, are warranted [6].

Learning in the flipped classroom has a number of potential benefits, including the implication that more responsibility for learning is transferred to the student. The optimal level of autonomy and flexibility in the flipped classroom, has been little studied [7]. It has been suggested that certain designs of the flipped classroom may promote self-regulated learning and higher-order thinking skills such as applying, analysing, evaluating and creating knowledge [8–11]. Another benefit of the flipped classroom model is that educators can be given more flexibility to cover a wider range and depth of material, as well as offer timely feedback and supervision to the students [11].

In designing flipped classroom approaches there are a number of factors to consider. It has been suggested that the principles for designing a flipped classroom approach include engaging students in self-learning at home, designing learning activities based on authentic problems, designing learning activities to engage students in higher-order thinking, encouraging peer-to-peer and peer-to-teacher interactions [12]. Due to the typical collaborative design of learning activities in the flipped classroom, designers should consider the affective dimensions of learning, including commitment to peers, being recognized, feeling safe, and the relationship with the educator were conducive for students’ learning [13].

Physiotherapists are increasingly working in settings where they take autonomous decisions that may place increased demands on team-working abilities. In addition, there is a global trend of a rising numbers of individuals with a range of disorders that largely cause disability but not mortality [14]. Physiotherapy education has a curriculum consisting mainly of theory (usually taking place at the university) and practice (usually taking place outside the university). The entry requirements of Physiotherapy programmes are usually relatively high, suggesting that the students who are admitted have already developed successful learning strategies. In order to graduate physiotherapy students who are able to thrive in increasingly complex health systems, it has been argued that educators must move away from teaching and learning strategies that disempower students and lecturers [15]. Until know, educational interventions that combine digital technology and active learning, have been little investigated within physiotherapy education.

The aim of this study was to describe the design, implementation and evaluation of a flipped classroom teaching approach in Physiotherapy education.

## Methods

The study design was a historically controlled, prospective, cohort study. The intervention took place within a second-year course on musculoskeletal disorders in the Bachelor Programme in Physiotherapy at OsloMet – Oslo Metropolitan University. Altogether, 54 students participated in the course which took place over eight weeks in autumn, 2017. The research project was registered with the Norwegian Centre for Research Data NSD (Ref: 55901/3/ STM). Three months before the course began, students were provided with information about the flipped classroom model and the associated implications on their learning, and then again a week before the course started. In order to clarify their expectations about work intensity outside the classroom, about 80 h on the students’ timetable were allocated for pre-classroom studying and after-class work.

### Development of the flipped classroom approach

The design of the flipped classroom approach was inspired by social constructivism, which emphasizes the importance of the learner being actively involved in the learning process and by literature on constructive course alignment [16–18]. The course-leader (YR) and the other educators involved in the course only had the minimum pedagogical requirements for teaching in higher education.

The pre-class learning resources consisted of about 12 h of pre-recorded video lectures, YouTube-videos, podcasts and an e-learning course. In addition, several key scientific papers, were included. The video lectures were produced by five educators, who had several years of experience with teaching. The lectures were recorded using the Microsoft OfficeMix platform, which allows an image- and audio track, parallel with PowerPoint slides. Typically, the digital lectures would include audio on all slides and a video of the educator on the first and last slides. The video lectures were accessible on all types of devices, including mobile phones. The digital learning resources were organised in seven themes (Table 1) and made available for the students a week in advance of the course.

**Table 1:** Overview of the in-class learning activities of the 8 weeks flipped classroom approach

Name of seminar and central themes	Learning resources
Standardized measures for musculoskeletal disorders: ✓ Different types of measures ✓ Quality criteria ✓ The International Classification of Functioning, Disability and Health (ICF)	• 95 min pre-recorded video lectures • Webpages and blogposts • YouTube videos • Book chapters and three scientific papers
Evidence-based practice (I and II) ✓ Model for evidence based practice ✓ The hierarchy of evidence ✓ Planning of treatment ✓ How to structure the patient journal ✓ *Self*-management interventions	• 75 min pre-recorded video lectures • An online course • YouTube videos • Webpages • Podcast episodes • Three scientific papers
Pain as an unpleasant sensory and emotional experience ✓ Nociception and pain processing ✓ Hypersensitivity ✓ Patient experiences with persistent pain ✓ Pain monitoring models ✓ Pharmacology and pain	• 174 min pre-recorded video lectures • An online course in patient education • YouTube videos • Webpages • Podcast episode • Seven scientific papers
Physiotherapy for upper-extremity disorders ✓ Epidemiology ✓ Diagnostic classification ✓ Prognostic factors ✓ Treatment	• 167 min pre-recorded video lectures • YouTube videos • Webpages and blogposts • Book chapters and one scientific paper
Physiotherapy for lower-extremity disorders ✓ Epidemiology ✓ Diagnostic classification ✓ Prognostic factors ✓ Treatment	• 83 min pre-recorded video lectures • YouTube videos • Book chapters and one scientific paper
Physiotherapy for low back pain ✓ Epidemiology ✓ Diagnostic classification ✓ Prognostic factors ✓ Treatment	• 128 min of pre-recorded video lectures • YouTube videos • Podcast episodes • Book chapters and four scientific papers
Generic learning resources	• Book chapters and five web-pages

The in-class learning activities consisted of seven full-day seminars held during a eight week period. In the seminars, the students worked on assignments in groups of about seven. In order to facilitate accountability and regulation of working, groups were constant throughout seminars. The central themes, sub-themes and learning resources for each seminar, are shown in Table 1. The assignments ranged in difficulty, from lower order thinking skills, to higher order [8]. Effort was made, to develop assignments which reflected authentic problems in physiotherapy practice [12]. For example, in one of the seminars (Evidence-based physiotherapy II), an assignment provided the students with a link to an animation film on healthcare professionals’ use of metaphors in communication with patients at a hospital. After seeing the film, the students were encouraged to identify similar types of metaphors in physiotherapy practice and discuss the consequences. In addition to the film, the learning resources included a link to a physiotherapy podcast were the use of metaphors was discussed. While some assignments were typically fact-based, others would require that the students critically debated a topic, using different perspectives.

All seminars had a similar structure, starting with a plenary session of about 45 min, where the students had the possibility to ask questions related to the pre-classroom digital learning resources. The plenary session was followed by five hours (including lunch and breaks), where the students were working in groups, solving assignments. The end of the seminars was devoted to student presentations where two groups exchanged and discussed answers of the assignments. The groups were encouraged to work on a shared document, for example in Google Drive or Microsoft OneDrive. This was supported by a study in a physiotherapy department, which found that using technology to engage in shared learning experiences facilitated the development of critical attitudes towards knowledge [19]. The use of fixed groups was inspired by a theory of team-based learning, groups were strategically formed featuring permanent teams with about seven members [20]. Because students’ responsibility for their own learning was a vital part of the approach, the in-classroom activities were non-mandatory and class attendance not systematically registered. However, the students were strongly encouraged to attend all face-to-face seminars.

The role of the educators was to council the groups and to organise the seminars. For each seminar at least two educators who were experts on the central themes, participated. These educators were usually the same ones who had recorded the video lectures that were watched by students at home. The participating educators had no previous experience with the flipped classroom model. Due to practical reasons, the educators did not go through any training with respect to the teaching role, before the course.

The course-exam took place about eight weeks after the last seminar. In the period between the last seminar and the course exam, the students were in clinical education. The examination was based on the assignments the students had worked on during the seminars, of which some were fact-based while other reflected higher order thinking. The topics of the assignments were consistent with those covered in Table 1. Altogether, eight assessors, working in pairs, participated in the exam. In order to increase reliability, the pairs of assessors were rotated during the day of examination. The decision with respect to grades was based on agreement between assessors, using the qualitative criteria decided by the Norwegian Association of Higher Education Institutions (UHR) (Table 2). In a preparatory meeting held for assessors, the criteria were discussed in detail. In addition, the importance of implementing both fact-based- and reasoning-based assignments in the examination, was emphasised. Agreement between assessors was not systematically assessed.

**Table 2:** General qualitative descriptions of grades in Norwegian higher education

Letter grades	Criteria used in the assessment of examinations.
A – Excellent	An excellent performance, clearly outstanding. The candidate demonstrates excellent judgement and a high degree of independent thinking
B – Very good	A very good performance. The candidate demonstrates sound judgement and a very good degree of independent thinking.
C – Good	A good performance in most areas. The candidate demonstrates a reasonable degree of judgement and independent thinking in the most important areas.
D – Satisfactory	A satisfactory performance, but with significant shortcomings. The candidate demonstrates a limited degree of judgement and independent thinking.
E – Sufficient	A performance that meets the minimum criteria, but no more. The candidate demonstrates a very limited degree of judgement and independent thinking.
F – Fail	A performance that does not meet the minimum academic criteria. The candidate demonstrates an absence of both judgement and independent thinking.

### Material and analyses

The learning outcome of the teaching approach was assessed with student performance on the course-exam and students’ perceptions. Student performance was assessed with the grades from the course-exam in 2017 and then compared with historical controls of conventional teaching practices in the same course from 2016, 2015, 2014 and 2013. The historical cohorts were similar prior to taking the course. The course grades were collapsed into three categories: Excellent, Very good and Good (A, B, and C), Satisfactory and Sufficient (D and E), and Fail (F). Due to the variation of the number of attendees in the courses over the historical period (range 49–60), only relative numbers (%), are reported, using descriptive statistics.

Student perceptions with respect to the teaching approach was measured in a survey that was distributed to the students after the last seminar. The survey contained six open-ended questions; whether they preferred flipped classroom teaching compared to conventional teaching, their preference for constant or movable groups, their satisfaction with the autonomy provided to them, the factors that either facilitated or hampered their learning using the flipped classroom approach, preparation for the seminars, and any other comments related to the approach. The open-ended survey questions were ordered in analytic categories, using a thematic analysis approach [21]. The analytical process included identifying and analysing themes within the data, following the stepwise guide from Braun and Clarke [21]. First we familiarised ourselves with the data. Next, we generated initial codes, and searched for themes. Lastly, we defined and named the themes. This analytical process was carried out by two of the authors (YR and TD-M). All authors participated in the final discussion of the analysis that took place in an online meeting.

## Results

**Fig. 1:**
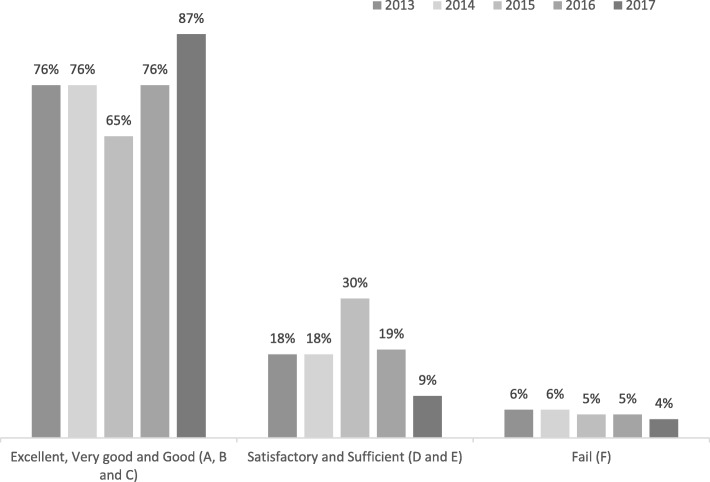
Relative distribution (%) of Excellent, Very good and Good (A, B and C), Satisfactory and Sufficient (D and E), and Fail (F) performances in the course exam of the years 2013–2017

At the final course-exam, 51 students passed, 2 failed and 1 did not attend (*n* = 54). Relative distributions of performance the year of the study (2017) as compared with the previous four years, are shown in Fig. 1 below. The share of students with Excellent, Very good and Good (A, B and C) performances showed a higher rate by more than 10% relative to any previous year. In addition, Satisfactory, Sufficient and Fail performances (D, E and F) showed a lower rate by more than 10%.

Altogether 46 students (87%) responded to the survey. Of these, 29 students reported that they preferred the flipped classroom approach in comparison to conventional teaching, 8 preferred conventional teaching and 9 reported that they were “not sure”.

With respect to the flexibility inherent the in-class activities, 20 students reported that they preferred a more structured classroom, 17 said that they were “not sure” and 9 students said that they preferred “as much autonomy as possible”. Furthermore, 35 students supported the decision to use fixed groups in the collaborative classroom activities, 8 preferred rotating group members, and 3 reported that they were “not sure”.

The most frequently reported factors that represent students’ view from the seminars were interaction with peers and educators, and the flexibility associated with digital learning resources (Table 3).

**Table 3:** Analytic categories representing students’ perceptions of the factors that influenced their learning at the seminars

	Examples from survey responses
Positive factors	
Interaction with peers and educators	*We can talk to and discuss with fellow students without interference*
Flexibility associated with digital learning resources	*Preparations can take place where and when we choose and in self-paced speed,*
Didactical aspects, such as course coherence, relevant assignments	*The exercises correspond with digital learning resources*
Negative factors	
Long and exhausting seminars	*Seminars took much time and often felt more exhausting than useful*
Little time for preparation or other reasons for not making preparations	*Heavy work-load in regard to preparation for the seminars*
Sub-optimal group dynamics or variation in ambitions among group members	*Difficult to have good discussion in the group because some members had not prepared*
Length of video lectures	*Some of the videos were much too long! You should take a look at the Khan academy!*
Lack of available educators during group work	*I wished there had been more discussions with the teacher during the group-work*

## Discussion

### Learning outcomes

The results of this flipped classroom teaching approach were promising with respect to students’ performance as assessed by the course-exam. Compared with historic controls, Excellent and Very good performances increased, while Satisfactory, Sufficient and Failed performances decreased. The results suggest that both students at the higher and lower levels of performance profited from the approach. However, there are at least two factors that should be considered in the interpretation of the results. Firstly, due the lack of a control group it is not possible to decide whether the observed effects were caused by other factors. For example, this may have been an exceptional student cohort. In addition, there were other changes made to the programme that may also have contributed to the improved outcomes, such as the authentic, group-based classroom activities that were not necessarily part of the flipped classroom approach. Furthermore, it cannot be completely ruled-out that the students modified their learning-behaviour in response to their awareness of being observed (Hawthorne-effect). Finally, the reliability and validity of course-grades as a measure for learning outcomes, is arguable. Nevertheless, considering these objections, we still think the results are important for educators who plan to alter their teaching methods in an attempt to develop higher order thinking skills in physiotherapy students. The improved grades observed in the present study are similar to findings in other studies: The flipping of an Engineering course resulted in improved performance (quiz, exam questions and open-ended design problems) and also allowed the educator to cover more material the first of these studies, students reported a preference for working in the smaller-class format in teams and also achieved significantly better course grades [22]. Similar findings were made in a study in Pharmacy education, where flipping of a large self-care course resulted in better overall course grades and improved opportunities to develop verbal communication skills and tackle unfamiliar problems [23].

It has been shown that flipped classroom teaching has the potential to enhance higher-order thinking skills and self-regulated learning, among students [10, 11, 22, 24]. Although we did no systematic investigation of this, the assessors at the course exam thought that the students had been able to discuss and debate at a higher level than in previous courses. As has already been mentioned, effort had been made to develop assignments which reflected higher-order thinking skills. This is, however, not unique for flipped classroom teaching, and similar approaches to learning can be implemented in other types of teaching. Nevertheless, we would strongly argue that an important success factor for these approaches to learning is the preparation made by students before attending the classroom, which is a key component of the flipped classroom model [2]. We also think that the collaborative working environment at the seminars, was imperative for the learning. Although the flipped classroom model give no directions with respect to learning activities, there is evidence that support the use of collaborative learning activities [25]. However, less is known with respect to the design of the learning activities. Guided by the literature on team-based learning, we chose to keep groups stable, through all the seminars [20]. We also hoped this positively would influence affective dimensions of learning, such as commitment to peers, being recognized and feeling safe, were acknowledge [13]. Results from the survey show that the choice of stable groups were supported by an overwhelming majority of the students.

In the survey, about two thirds of the students reported that self-perceived learning outcomes of the flipped classroom approach was superior to previous conventional teaching in the programme. These results are in line with a systematic review in medical education which concluded that the flipped classroom is a promising teaching approach to increase learners’ motivation and engagement [26].

Physiotherapy education should reflect present and future demands of the health care. Due to the global rising numbers of individuals with a range of disorders that largely cause disability rather than mortality, patient education strategies are increasingly emphasized, within the context of rehabilitation [14, 27, 28]. While physiotherapy education has traditionally focused on physical activity, exercises and manual skills, future education will need to expand learning with respect to communication, critical thinking and collaboration, within a clinical setting. The flipped classroom model represent an opportunity to implement higher-order learning skills in the teaching. Nevertheless, yet there is only rudimentary evidence that blended learning has the potential to improve clinical competencies among health students [29].

### Lessons learned and future developments

One of the strengths of this teaching approach is that it offers students a well-planned, flexible and coherent working process. The survey responses indicate that although some students enjoyed the flexibility and autonomy of the preparatory work, about half would prefer firmer structure in the seminars (Table 3). In retrospect, we think that this preference can be explained by the fact that the students’ previous experience in higher education is strongly associated with fact-based courses like anatomy, biomechanics and physiology. Asking students to shift into a new learning paradigm, when their previous learning strategies have been successful, may be a challenge for some. This is also supported by responses from the survey, were almost half of students reported that they preferred more structured classroom activities. Few studies have investigated the optimal degree of flexibility that is associated with this type of learning [7].

In order to increase students’ responsibility for their own learning, the seminars for this course were non-mandatory and class-attendance was not systematically recorded. We anticipated that group-accountability would be a barrier for absence. Although instructors never observed significantly low attendance, we do not know whether some students were absent from several seminars, or whether students who did not attend seminars performed poorly in the course-exam. In retrospect, we think that group-accountability alone not necessarily ensure class attendance. There were some indications in the survey responses that all groups did not work optimally. Our aim for future courses is to keep the learning activities non-mandatory. Instead, learning activities can be structured differently. For example, an assessed element at the end of group-work, which contributed to the final exam, could have been implemented. Another step would be to have students anonymously assessing other group-members’ contributions.

Findings in a study on the flipped classroom showed that there was a tendency among students to regard class attendance as optional, as there was a perception that complete learning could be achieved by viewing video lectures alone [30]. The present approach The development of the learning activities were informed by social constructivism, which emphasizes the importance of the learner being actively involved in the learning process [16]. Furthermore, the course-design was inspired by theories of constructive alignment. The basic premise of constructive alignment is that the curriculum is designed so that the learning activities and assessment tasks are aligned in order to support students to attain the goals intended for the course [18].

Without doubt, the in-classroom learning activities in the present study offered students too little variation. “Long and exhausting seminars” was the most common complaint from survey respondents. Due to this, we would like to increase variation in the feedback sessions for future courses. Example of activities that could have added variation are facilitator-led discussions and poster-presentations. We also think that there is some potential for implementing digital, interactive, learning activities and social-media platforms in the learning activities, which may facilitate remote interaction [10]. Furthermore, research from the fields of human memory and recall has claimed that learning is better achieved when spaced out over time, in smaller chunks. In support of this, another solution could be to break the session up into different periods of the day, or even extend it over a period of a week [31].

It has been advocated that the role of the educator in the flipped classroom should be active, rather than passive [10]. As could be expected, there are indications that educators who have previous experiences with active learning, more easily adapt to teaching in the flipped classroom [32]. In the present teaching approach, educators had little previous experience, nor received any training. There is some indications from the survey responses, that students would have preferred increased availability of educators during the group work. However, responses also indicate that students appreciated autonomous discussion with their peers, with the option to contact educators, if necessary. In retrospect, we think training of the educators before the teaching approach would have helped the educators to find an optimal level of activity. However, at the time the design of the intervention took place, much effort was devoted to the technical issues concerning the production of video lectures.

### Study limitations

As has already been mentioned, this study had some limitations with respect to the interpretation of the improved learning outcomes. The study design did not control for external factors that may have affected students’ performance relative to previous cohorts. It is also worth considering that a well-planned, conventional learning environment, may also lead to the kinds of improvements in learning outcomes that were observed during this study. Nevertheless, we would argue that an important success-factor lies in the combined effect of the preparatory work and the well-organised, collaborative learning activities.

## Conclusions

A flipped classroom approach in physiotherapy education resulted in improved student performances in this professional programme, when compared with historical control of conventional teaching. Students responded positively to the collaborative learning environment, especially with respect to the associated autonomy and flexibility. There was indication that all groups did not work optimally and that accountability to other group members did not always ensure pre-classroom preparations. The results indicate that physiotherapy students benefit from student-focused teaching strategies, associated with increased responsibility of learning. Further research is warranted to investigate whether health professions students are able to profit from this type of learning in a clinical setting, as well as to determine the optimal level of autonomy and flexibility in a flipped classroom approach.

## Data Availability

Datasets supporting the conclusions of this article are included within the article. Additional data at the level of individual students or educators are not available as per confidentiality agreements approved by the Norwegian Centre for Research Data.
